# Relative Burden of Cancer and Noncancer Mortality Among Long-Term Survivors of Breast, Prostate, and Colorectal Cancer in the US

**DOI:** 10.1001/jamanetworkopen.2023.23115

**Published:** 2023-07-12

**Authors:** Madhav KC, Jane Fan, Terry Hyslop, Sirad Hassan, Michael Cecchini, Shi-Yi Wang, Andrea Silber, Michael S. Leapman, Ira Leeds, Stephanie B. Wheeler, Lisa P. Spees, Cary P. Gross, Maryam Lustberg, Rachel A. Greenup, Amy C. Justice, Kevin C. Oeffinger, Michaela A. Dinan

**Affiliations:** 1 Yale Cancer Outcomes, Public Policy, and Effectiveness Research Center, New Haven, Connecticut; 2 Department of Biostatistics, Yale School of Public Health, New Haven, Connecticut; 3 Sidney Kimmel Cancer Center, Thomas Jefferson University, Philadelphia, Pennsylvania; 4 Section of Medical Oncology, Department of Internal Medicine, Yale University School of Medicine, New Haven, Connecticut; 5 Department of Chronic Disease Epidemiology, Yale School of Public Health, New Haven, Connecticut; 6 Department of Internal Medicine, Yale University School of Medicine, New Haven, Connecticut; 7 Department of Urology, Yale University School of Medicine, New Haven, Connecticut; 8 Department of Surgery, Yale University School of Medicine, New Haven, Connecticut; 9 Department of Health Policy and Management, Gillings School of Global Public Health, University of North Carolina at Chapel Hill; 10 Lineberger Comprehensive Cancer Center, University of North Carolina at Chapel Hill; 11 Department of Population Health Sciences, Duke University, Durham, North Carolina; 12 Duke Cancer Institute, Duke University, Durham, North Carolina

## Abstract

**Importance:**

Improvements in cancer outcomes have led to a need to better understand long-term oncologic and nononcologic outcomes and quantify cancer-specific vs noncancer-specific mortality risks among long-term survivors.

**Objective:**

To assess absolute and relative cancer-specific vs noncancer-specific mortality rates among long-term survivors of cancer, as well as associated risk factors.

**Design, Setting, and Participants:**

This cohort study included 627 702 patients in the Surveillance, Epidemiology, and End Results cancer registry with breast, prostate, or colorectal cancer who received a diagnosis between January 1, 2003, and December 31, 2014, who received definitive treatment for localized disease and who were alive 5 years after their initial diagnosis (ie, long-term survivors of cancer). Statistical analysis was conducted from November 2022 to January 2023.

**Main Outcomes and Measures:**

Survival time ratios (TRs) were calculated using accelerated failure time models, and the primary outcome of interest examined was death from index cancer vs alternative (nonindex cancer) mortality across breast, prostate, colon, and rectal cancer cohorts. Secondary outcomes included subgroup mortality in cancer-specific risk groups, categorized based on prognostic factors, and proportion of deaths due to cancer-specific vs noncancer-specific causes. Independent variables included age, sex, race and ethnicity, income, residence, stage, grade, estrogen receptor status, progesterone receptor status, prostate-specific antigen level, and Gleason score. Follow-up ended in 2019.

**Results:**

The study included 627 702 patients (mean [SD] age, 61.1 [12.3] years; 434 848 women [69.3%]): 364 230 with breast cancer, 118 839 with prostate cancer, and 144 633 with colorectal cancer who survived 5 years or more from an initial diagnosis of early-stage cancer. Factors associated with shorter median cancer-specific survival included stage III disease for breast cancer (TR, 0.54; 95% CI, 0.53-0.55) and colorectal cancer (colon: TR, 0.60; 95% CI, 0.58-0.62; rectal: TR, 0.71; 95% CI, 0.69-0.74), as well as a Gleason score of 8 or higher for prostate cancer (TR, 0.61; 95% CI, 0.58-0.63). For all cancer cohorts, patients at low risk had at least a 3-fold higher noncancer-specific mortality compared with cancer-specific mortality at 10 years of diagnosis. Patients at high risk had a higher cumulative incidence of cancer-specific mortality than noncancer-specific mortality in all cancer cohorts except prostate.

**Conclusions and Relevance:**

This study is the first to date to examine competing oncologic and nononcologic risks focusing on long-term adult survivors of cancer. Knowledge of the relative risks facing long-term survivors may help provide pragmatic guidance to patients and clinicians regarding the importance of ongoing primary and oncologic-focused care.

## Key Points

**Question:**

What is the relative burden of oncologic and nononcologic mortality among long-term survivors of cancer in the US?

**Findings:**

In this cohort study of 627 702 patients surviving 5 years or more from an initial diagnosis of early-stage breast, prostate, or colorectal cancer, the risk of dying from the index cancer varied widely relative to noncancer-specific causes of death. Patients with low oncologic risk at the time of diagnosis had at least 3-fold higher risk of noncancer death compared with death from the index cancer.

**Meaning:**

This study suggests that risk-stratified care may help quantify the relative importance of oncologic and primary care surveillance for long-term survivors of cancer.

## Teaser

This cohort study assesses absolute and relative cancer-specific vs noncancer-specific mortality rates among long-term survivors of cancer, as well as associated risk factors.

## Introduction

Due to improvements in early detection, treatment, and oncologic outcomes, many survivors of cancer are now living longer and are thus more likely to experience or die from conditions other than their original cancer,^1,2,3,4^ with two-thirds of all patients with cancer now living 5 years or more after diagnosis.^5^ Risk-stratified models of care have emerged as a critical strategy that could be used to appropriately allocate care intensity between the oncologist and primary care physician (PCP)^6,7,8,9,10^ and has been highlighted by the American Cancer Society, the American Society of Clinical Oncology, and the National Cancer Institute as an area of priority research.^1,6,11^ These models emphasize coordination between oncologists and PCPs while accommodating the unique oncologic and nononcologic health needs of survivors of cancer^12,13,14^ and have the potential to dramatically reduce large-scale inefficiencies in care while improving the quality of care.^15^ Population-level studies have considered competing risks of cancer vs noncancer mortality in breast, prostate, and colorectal cancers^16,17,18,19,20^ and have helped provide insights into the relative association of each with mortality, but they have not focused on long-term (≥5 years) survivors of definitively treated disease.^21,22,23,24^ Long-term survivors should be studied to help inform the management of patients under surveillance by their oncologist who reach the 5-year mark and require pragmatic risk assessment in the upcoming years.

As such, there is a critical need to provide quantitative risk estimates of oncologic and nononcologic outcomes among long-term survivors of cancer in representative US cohorts. Given that breast, prostate, and colorectal cancers account for half of all diagnoses for survivors of cancer, individuals with these cancers provide the ideal study population for survivorship risk stratification research.^25,26^ The ability to directly inform survivorship care is hampered in that these studies (1) often included patients who did not undergo curative treatment and/or had metastatic disease, (2) were not focused on long-term survivors (ie, those surviving at ≥5 years from diagnosis), (3) did not attempt to differentiate factors associated with cancer-specific vs noncancer-specific mortality, and (4) did not define cancer-specific vs noncancer-specific events from an optimal surveillance perspective. As such, relevant empirical data are lacking to inform long-term survivors of these common cancers of their relative risk of cancer-specific vs noncancer-specific mortality, which could be used to help inform models of survivorship care that are tailored to patients’ unique risk profiles.

In this study, our objective was to assess the absolute and relative risks of cancer-specific vs noncancer-specific mortality among long-term (≥5 years) survivors of breast, prostate, colon, and rectal cancers within the US, to ultimately promote risk assessments to inform the implementation of risk-stratified survivorship pathways.

## Methods

### Data Source

We used data from the most recent data set (2021) of the National Cancer Institute’s Surveillance, Epidemiology, and End Results (SEER) program.^27^ The SEER program collects population-based cancer incidence and survival data across 18 registries in the US covering approximately 48.0% of the US population. The Yale institutional review board approved this study as exempt because SEER-Medicare data were deidentified, and informed consent was not required. This study followed the Strengthening the Reporting of Observational Studies in Epidemiology (STROBE) reporting guideline.

### Study Population

**Figure 1.**
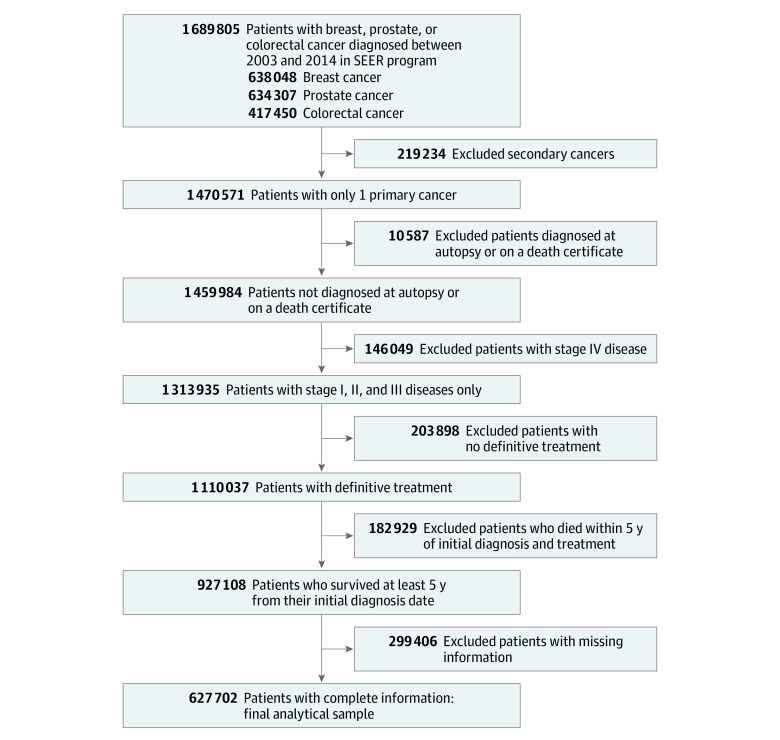
Flowchart of Inclusion and Exclusion Criteria SEER indicates Surveillance, Epidemiology, and End Results.

Patients who received a diagnosis of breast, prostate, or colorectal cancer from January 1, 2003, through December 31, 2014, with documented receipt of definitive treatment and who had survived at least 5 years from diagnosis were included. Definitive treatment was defined by site-directed surgery and, among patients with prostate cancer, also included radiotherapy. Patients with stage IV disease and those diagnosed at autopsy or on a death certificate were excluded. In addition, patients with missing information on demographic characteristics, clinical factors, or duration of follow-up were excluded (Figure 1; eMethods in Supplement 1).

### Measures

#### Study Outcomes

The outcomes of interest were cancer-specific mortality and noncancer-specific mortality. The outcomes were defined based on SEER cause-of-death classification variables.^28^ Mortality due to the primary cancer was classified as cancer-specific mortality. Mortality due to causes other than the primary cancer was classified as noncancer-specific mortality. Hence, for a patient whose index cancer was breast cancer, a death from any other cancer, as well as any noncancer condition, would be classified as noncancer-specific. The clinical motivation for the study was to prioritize the need for cancer-specific vs noncancer-specific (eg, primary care, cardiology, and pulmonology) clinician surveillance at 5 years after the diagnosis of a definitively treated, early-stage cancer. Although the 5-year benchmark is somewhat debatable, it remains a practical window after which nononcologic follow-up is considered for many cancers. For example, the National Institutes of Health have used a 5-year horizon in multiple requests for applications to investigate long-term survivors of cancer.^29^ Cancer-specific events were defined as death from the incident cancer, which would presumably be most effectively detected and/or managed by a patient’s initial oncologist.

#### Independent Variables

##### Demographic Factors

All demographic and clinical factors were collected at the time of diagnosis, including patients’ age, sex (colorectal cancer only), race and ethnicity (Hispanic, Non-Hispanic American Indian or Alaska Native, Non-Hispanic Asian or Pacific Islander, Non-Hispanic Black, and Non-Hispanic White), year of diagnosis, median household income (county level), and area of residency (metropolitan vs nonmetropolitan) and were included in the analysis. Race and ethnicity were classified by the SEER program. The SEER program determines race and ethnicity through medical record abstraction or by using a computer algorithm that searches surnames of the reported cases to determine Hispanic origin. Although certain cancer characteristics do vary across racial and ethnic groups, race and ethnicity were included in the study not as a biological construct, but as a proxy for structural racism. Age at diagnosis was categorized into 5 categories: younger than 50 years, 50 to 54 years, 55 to 59 years, 60 to 64 years, 65 years or older. All patients lived at least 5 years after diagnosis. Therefore, patients aged 65 years or older at the time of diagnosis were 70 years or older at their entry into the study.

##### Tumor- and Treatment-Related Factors

Tumor-related factors at the time of diagnosis included stage, grade, nodal status, hormone receptor (estrogen and progesterone) status (breast cancer only), laterality (breast cancer only), prostate-specific antigen (PSA) level (prostate cancer only), and Gleason score (prostate cancer only). We used American Joint Committee on Cancer staging reported in SEER for cancer stage. Grade IV disease was infrequently reported and therefore grouped with grade III. Prostate-specific antigen level was categorized as low (<10 ng/mL), intermediate (10-20 ng/mL), or high (>20 ng/mL) (to convert to micrograms per liter, multiply by 1.0). Gleason score was also categorized as low (≤6), intermediate (7), or high (≥8). Treatment-related factors included chemotherapy and radiotherapy.

### Statistical Analysis

Statistical analysis was conducted from November 2022 to January 2023. A least absolute shrinkage and selection operator (LASSO) was used to select the factors associated with cancer-specific and noncancer-specific mortality separately. Variables with a regression coefficient equal to zero after the shrinking process were excluded from the model, and variables with nonzero coefficients were included in survival analysis (eTable 3 in Supplement 1). Models estimating cancer-specific and noncancer-specific mortality for each cancer site were created. Due to differences in the epidemiology and treatment of rectal cancers, independent models for colon and rectal cancers were built.

Because the proportional hazards assumption was not satisfied for several covariates, we used accelerated failure time (AFT) models, which do not rely on the proportional hazards assumption.^30,31,32^ Parameter estimates from AFT models were converted to time ratio (TR) estimates to interpret the effect of a covariate on the time scale. A TR greater than 1 indicates that the covariate is associated with accelerated survival time (ie, longer median survival), whereas a TR less than 1 indicates that the covariate is associated with decelerated survival time (ie, shorter median survival).

Based on established risk factors of cancer-specific mortality, patients with cancer were grouped into 3 risk groups of oncologic mortality: low risk, intermediate risk, or high risk.^33,34,35,36,37^ For patients with breast cancer, risk groups were categorized as (1) low risk (≥65 years and stage I), (2) high risk (<65 years and stages II-III), or (3) intermediate risk (everyone else). Patients with prostate cancer were classified as (1) low risk (≥65 years and Gleason score of 6), (2) high risk (<65 years and Gleason score >6), or (3) intermediate risk (everyone else). Patients with colorectal cancer were classified as (1) low risk (≥65 years and stage I), (2) high risk (<65 years and stages II-III), or (3) intermediate risk (everyone else).

The cumulative incidence function curves of cancer-specific and noncancer-specific mortality by risk groups were generated for all cancer sites. Statistical analyses were conducted using SAS, version 9.4 (SAS Institute Inc), Stata, version 17 (StataCorp LP), and R, version 4.0.4 (R Group for Statistical Computing). All *P* values were from 2-sided tests and results were deemed statistically significant at *P* < .05. The package grpreg was used to perform LASSO regularization in R.

## Results

### Cohort Characteristics

We identified 627 702 patients (mean [SD] age, 61.1 [12.3] years; 434 848 women [69.3%]): 364 230 with breast cancer, 118 839 with prostate cancer, 104 488 with colon cancer, and 40 145 patients with rectal cancer with stage I, II, or III disease diagnosed between 2003 and 2014 and treated with definitive intent surgery and/or radiotherapy.

A total of 123 701 patients with breast cancer (34.0%), 57 958 patients with prostate cancer (48.8%), 56 839 patients with colon cancer (54.4%), and 15 464 patients with rectal cancer (38.5%) were aged 65 years or older (Table 1). A total of 446 058 patients (71.1%) across all cancer cohorts were non-Hispanic White. Tumor stage at diagnosis varied substantially by cancer site. Approximately 10% of patients with breast cancer (35 560 [9.8%]) and 16 030 patients with prostate cancer (13.5%) received a diagnosis of stage III disease, whereas 31 399 patients with colon cancer (30.0%) and 13 593 patients with rectal cancer (33.8%) received a diagnosis of stage III disease.

**Table 1.** Patients’ Demographic and Clinical Characteristics

Characteristic	Patients, No. (%)			
	Breast cancer cohort (n = 364 230)	Prostate cancer cohort (n = 118 839)	Colon cancer cohort (n = 104 488)	Rectal cancer cohort (n = 40 145)
Age, mean (SD), y	58.9 (12.8)	63.9 (8.0)	65.2 (13.1)	60.7 (12.3)
Age at diagnosis, y				
<50	92 634 (25.4)	4230 (3.6)	11 812 (11.3)	7035 (17.5)
50-54	48 570 (13.3)	10 660 (9.0)	10 521 (10.1)	6005 (15.0)
55-59	49 461 (13.6)	19 551 (16.5)	11 929 (11.4)	5889 (14.7)
60-64	49 864 (13.7)	26 440 (22.3)	13 387 (12.8)	5752 (14.3)
≥65	123 701 (34.0)	57 958(48.8)	56 839 (54.4)	15 464 (38.5)
Sex				
Female	364 230 (100.0)	0	53 490 (51.2)	17 128 (42.7)
Male	0	118 839 (100.0)	50 998 (48.8)	23 017 (57.3)
Race and ethnicity				
Hispanic	37 805 (10.4)	11 238 (9.5)	10 685 (10.2)	4472 (11.1)
Non-Hispanic American Indian or Alaska Native	1561 (0.4)	365 (0.3)	466 (0.5)	216 (0.5)
Non-Hispanic Asian or Pacific Islander	31 676 (8.7)	6052 (5.1)	8883 (8.5)	4108 (10.2)
Non-Hispanic Black	32 439 (8.9)	17 513 (14.7)	11 202 (10.7)	2963 (7.4)
Non-Hispanic White	260 749 (71.6)	83 671 (70.4)	73 252 (70.1)	28 386 (70.7)
Income, $				
<40 000	11 101 (3.1)	5426 (4.6)	4296 (4.1)	1670 (4.2)
40 000-54 999	59 766 (16.4)	23 878 (20.1)	19 897 (19.1)	7522 (18.7)
55 000-69 999	138 761 (38.1)	47 100 (39.6)	40 038 (38.3)	15 202 (37.9)
≥70 000	154 602 (42.4)	42 435(35.7)	40 257 (38.5)	15 751 (39.2)
Residence				
Metropolitan	38 371 (10.5)	13 653 (11.5)	14 291 (13.7)	5480 (13.7)
Nonmetropolitan	325 859 (89.5)	105 186 (88.5)	90 197 (86.3)	34 665 (86.3)
Stage				
I	197 245 (54.2)	20 294 (17.1)	33 715 (32.3)	15 723 (39.2)
II	131 425 (36.0)	82 515 (69.4)	39 374 (37.7)	10 829 (27.0)
III	35 560 (9.8)	16 030 (13.5)	31 399 (30.0)	13 593 (33.8)
Grade				
I	86 628 (23.8)	NA	12 339 (11.8)	11.8 (12.0)
II	160 200 (44.0)	NA	75 292 (72.1)	30 588 (76.2)
III	117 402 (32.2)	NA	16 857 (16.1)	4736 (11.8)
ER status				
Negative	62 333 (17.1)	NA	NA	NA
Positive	301 897 (82.9)	NA	NA	NA
PR Status				
Negative	99 462 (27.3)	NA	NA	NA
Positive	264 768 (72.7)	NA	NA	NA
PSA, ng/mL				
<10	NA	93 608 (78.8)	NA	NA
10-20	NA	17 690 (14.9)	NA	NA
>20	NA	7541 (6.3)	NA	NA
Gleason score				
≤6	NA	48 065 (40.5)	NA	NA
7	NA	52 797 (44.4)	NA	NA
≥8	NA	17 977 (15.1)	NA	NA

Abbreviations: ER, estrogen receptor; NA, not applicable; PR, progesterone receptor; PSA, prostate-specific antigen.

SI conversion factor: To convert PSA to micrograms per liter, multiply by 1.0.

Among patients with breast cancer, 301 897 (82.9%) were estrogen receptor (ER) positive and 264 768 (72.7%) were progesterone receptor positive (Table 1). A total of 7541 patients with prostate cancer (6.3%) had a PSA level higher than 20 ng/mL, and 17 977 (15.1%) had a Gleason score of 8 or higher.

### Noncancer-Related Causes of Death

Across all cancer cohorts, most patients died of noncancer-related causes. Heart disease was the leading cause of noncancer-specific deaths, followed by Alzheimer disease, chronic obstructive pulmonary disease (COPD), and cerebrovascular disease (eTable 1 in Supplement 1). Heart disease accounted for more than one-fourth of all noncancer-related deaths.

In the breast cancer cohort, two-thirds of patients died of causes other than their primary cancer, of which 24.0% (9210 of 38 348) were associated with heart disease (eTable 1 in Supplement 1). After heart disease, Alzheimer disease (7.1% [2727 of 38 348]), cerebrovascular diseases (6.6% [2522 of 38 348]), and COPD (6.5% [2474 of 38 348]) were also the common causes of noncancer-specific deaths among patients with breast cancer. In the prostate cancer cohort, 77.9% of total deaths (7179 of 9220) were noncancer specific; heart disease (24.5% [1758 of 7179]) was the most common cause of noncancer-related deaths, followed by COPD (6.1% [441 of 7179]), cerebrovascular diseases (4.8% [343 of 7179]), and Alzheimer disease (3.5% [249 of 7179]).

Among patients with colorectal cancer, more than two-thirds died of noncancer-related causes, of which almost one-third were associated with heart disease (eTable 1 in Supplement 1). The other common causes of noncancer-specific deaths included COPD, cerebrovascular diseases, and Alzheimer disease.

### Factors Associated With Cancer-Specific and Noncancer-Specific Mortality

**Figure 2.**
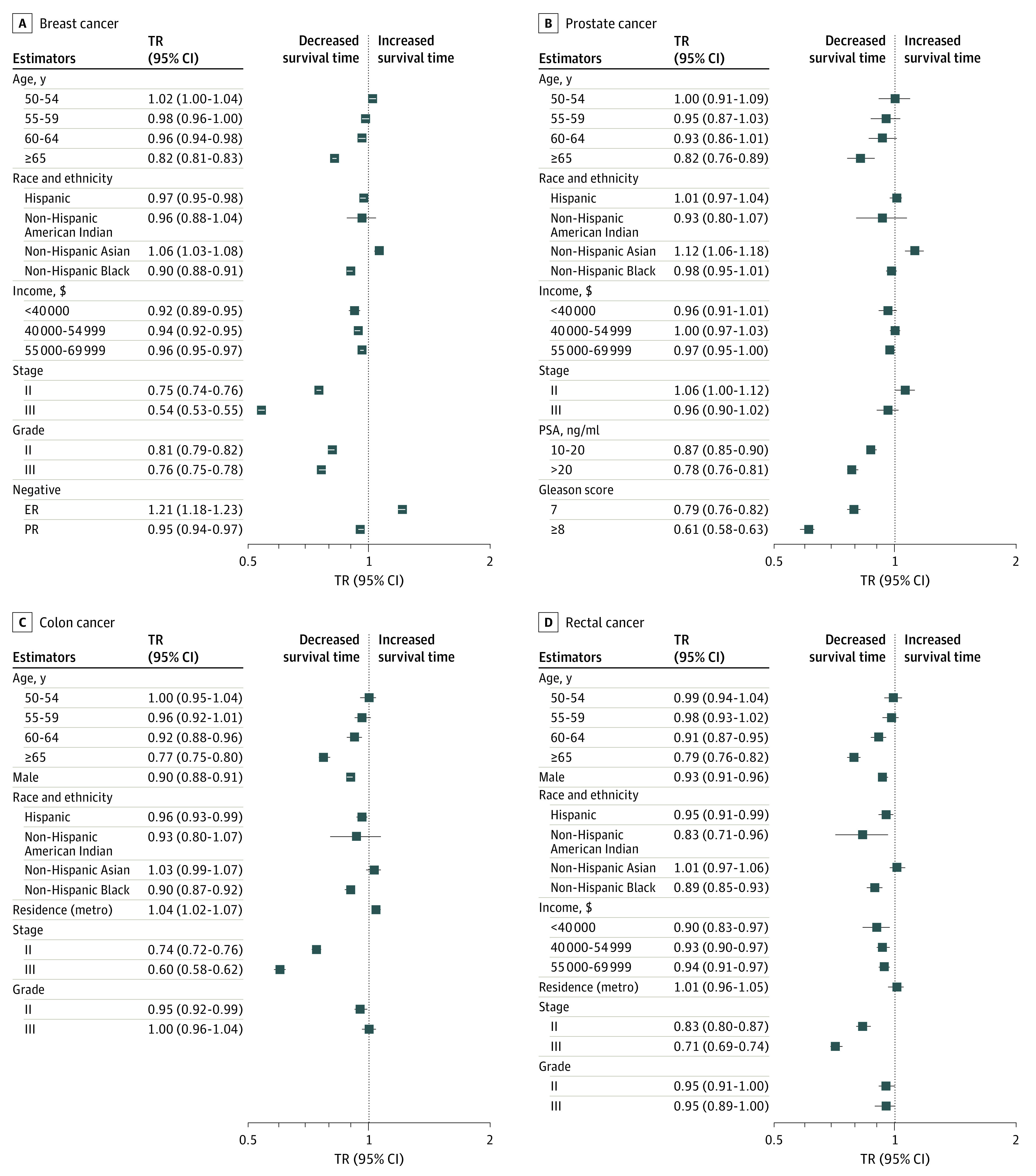
Survival Time Ratio (TR) of Cancer-Specific Mortality in Breast, Prostate, Colon, and Rectal Cancer Cohorts ER indicates estrogen receptor; metro, metropolitan area; PR, progesterone receptor; and PSA, prostate-specific antigen. To convert PSA to micrograms per liter, multiply by 1.0.

Patients with stage III breast cancer had a 46% reduction in median survival time for breast cancer–specific mortality than those with stage I disease (TR, 0.54; 95% CI, 0.53-0.55). Likewise, patients with grade III breast cancer had a 24% reduction in median survival time for breast cancer–specific mortality than those with grade I disease (TR, 0.76; 95% CI, 0.75-0.78) (Figure 2). Patients with stage III breast cancer had a 19% (TR, 0.81; 95% CI, 0.79-0.82) reduction in median survival time for noncancer-specific mortality, and those with grade III breast cancer had a 2% (TR, 0.98; 95% CI, 0.97-0.99) reduction in median survival time for noncancer-specific mortality (eFigure 1 in Supplement 1).

In the prostate cancer cohort, patients with a PSA level higher than 20 ng/mL had a 22% reduction in median survival time for prostate cancer–specific mortality (TR, 0.78; 95% CI, 0.76-0.81), and those with a Gleason score of 8 or higher had almost a 40% reduction in median survival time for prostate cancer–specific mortality (TR, 0.61; 95% CI, 0.58-0.63) (Figure 2). Patients with a PSA level higher than 20 ng/mL had an 11% (TR, 0.89; 95% CI, 0.88-0.91) reduction in median survival time for nonprostate cancer–specific mortality, and those with a Gleason score of 8 or higher had a 13% (TR, 0.87; 95% CI, 0.85-0.88) reduction in median survival time for nonprostate cancer–specific mortality (eFigure 1 in Supplement 1). Patients with stage III colon cancer, compared with stage I, had a 40% (TR, 0.60; 95% CI, 0.58-0.62) reduction in median survival time for colon cancer–specific mortality and an 8% (TR, 0.92; 95% CI, 0.91-0.93) reduction in median survival time for noncolon cancer–specific mortality (Figure 2; eFigure 1 in Supplement 1). Compared with stage I rectal cancer, patients with stage III disease had a 29% reduction in median survival time for rectal cancer–specific mortality (TR, 0.71; 95% CI, 0.69-0.74) (Figure 2).

### Cancer-Specific vs Noncancer-Specific Cumulative Mortality by Risk Group

**Figure 3.**
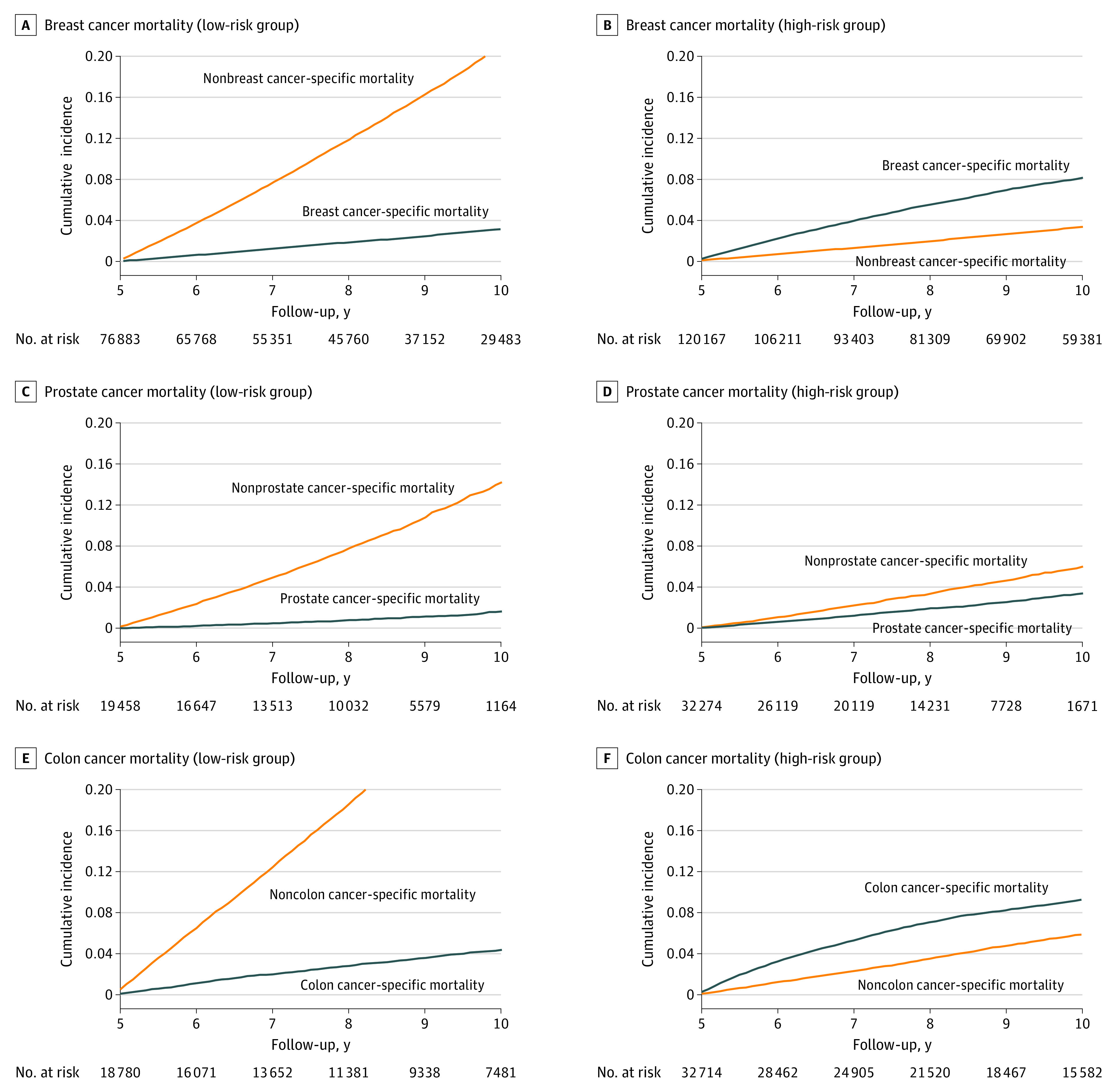
Cumulative Mortality for Patients With Breast, Prostate, and Colon Cancer by Risk Groups

Based on established risk factors of cancer-specific mortality, which were also confirmed by our analysis, patients were categorized into 3 risk groups. After 10 years of cancer diagnosis, there was a substantially different risk of cancer-specific vs noncancer-specific mortality between the low-risk and high-risk groups (Figure 3; eFigure 2 in Supplement 1). For patients with breast cancer in the low-risk group, defined as those 65 years or older and with stage I disease, the cumulative incidence of nonbreast cancer–specific mortality was almost 7 times higher than the cumulative incidence of breast cancer–specific mortality (Table 2). However, patients in the high-risk group, defined as those younger than 65 years and with stage II to III disease, had almost 2.5 times higher breast cancer–specific mortality than nonbreast cancer–specific mortality.

**Table 2.** Cumulative Incidence of Noncancer- and Cancer-Specific Mortality After 10 Years of Initial Diagnosis

Cancer type and risk group	Criteria	Cumulative incidence of noncancer-specific mortality (%)	Cumulative incidence of cancer-specific mortality (%)	*P* value	Cumulative mortality ratio (noncancer- vs cancer-specific mortality)
Breast cancer					
Low	≥65 y and Stage I	20.9	3.2	<.001	6.7
Intermediate	Everyone else	8.5	4.2	<.001	2.0
High	<65 y and Stages II-III	6.0	8.1	<.001	0.4
Prostate cancer					
Low	≥65 y and Gleason score ≤6	14.2	1.7	<.001	8.6
Intermediate	Everyone else	11.9	3.2	<.001	3.7
High	<65 y and Gleason score >6	6.0	3.4	<.001	1.8
Colon cancer					
Low	≥65 y and Stage I	30.6	4.4	<.001	7.0
Intermediate	Everyone else	23.5	8.0	<.001	3.0
High	<65 y and Stages II-III	5.9	9.3	<.001	0.6
Rectal cancer					
Low	≥65 y and Stage I	26.5	8.5	<.001	3.1
Intermediate	Everyone else	15.4	10.2	<.001	1.5
High	<65 y and Stages II-III	5.6	13.5	<.001	0.4

Among patients with prostate cancer, the low-risk group had an almost 9 times higher cumulative incidence of nonprostate cancer–specific mortality compared with the cumulative incidence of prostate–specific mortality (Table 2). The cumulative incidence of nonprostate cancer–specific mortality was also 1.8 times higher than the cumulative incidence of prostate cancer–specific mortality among those in the low-risk group, defined as those 65 years or older and with a Gleason score of 6 or lower. The cumulative incidence of noncancer-specific mortality among the low-risk colon and rectal cancer cohorts was 7 times and 3 times higher than cancer-specific mortality, respectively.

## Discussion

Estimating the relative risk of cancer-specific vs noncancer-specific mortality among long-term survivors of cancer is a critical first step in the development of risk-stratified models of care. Although many studies have previously examined competing oncologic vs nononcologic risks of common cancers,^16,17,18,19,20,21,22,23,24,29,38^ to our knowledge, this study is the first to focus on long-term (≥5 years) survivors. We found that the risk of oncologic and nononcologic mortality among long-term survivors of cancer varied widely by risk group. Ten years after cancer diagnosis, the noncancer-specific mortality was substantially higher than the cancer-specific mortality among patients with low oncologic risk, as assessed using standard prognosticating markers including stage or Gleason score. Conversely, cancer-specific mortality was high among those with adverse prognostic factors for their cancer (ie, high oncologic risk), except patients with prostate cancer. By quantifying the relative long-term risks of oncologic vs nononcologic mortality among these patients, we hope to help patients and clinicians place the relative importance of oncologic and nononcologic care into perspective. Although defining risk-stratified management is beyond the scope of this study, our findings suggest that patient groups with relatively high risks of nononcologic mortality, such as those 65 years or older with lower-stage disease, may particularly benefit from higher-intensity primary care surveillance. Given that the benefit associated with preventive care takes years to manifest, increased intensity of primary care may be most effective if initiated shortly after diagnosis, which could take place concurrently with oncologic management.

The factors associated with mortality among long-term survivors varied depending on patients’ age, tumor biology, and stage at diagnosis. Patients with low oncologic risk—defined as those aged 65 years or older, with a low tumor stage, and with a low Gleason score (for prostate cancer)—had substantially higher mortality associated with causes other than their initial cancer. Heart disease, Alzheimer disease, COPD, cerebrovascular disease, and lung disease were the dominant causes of death among patients with low oncologic risk across all cancer sites. In the low oncologic risk group, the ratio of cumulative mortality of noncancer-specific vs cancer-specific causes of death between 5 and 10 years after diagnosis was highest in the prostate cancer cohort (9-fold) followed by the colon (7-fold), breast (7-fold), and rectal (3-fold) cancer cohorts.

Previous studies have suggested that high-risk biology and stage at diagnosis increase the risk of cancer-related mortality among young women with breast cancer.^20,33,36,37,39,40,41,42^ For example, a recent study using SEER data found a greater risk of breast cancer–specific mortality among younger women with more advanced and aggressive disease than older women with hormone receptor–positive and low-grade breast cancer, but that age was not independently associated with an increased risk of mortality for other tumor subtypes.^43^ However, such studies were not restricted to long-term survivors. In our cohort of long-term survivors, we found that older age (ie, ≥60 years) was associated with poor cancer-specific survival. This finding is consistent with longer-term studies of breast cancer, which have reported an increased risk of cancer-specific mortality among older women compared with younger women.^44,45,46,47^ Our study used 5 years as the definition of long-term survivors. Other definitions of survivorship windows have been reported,^48^ and one could consider using different definitions for different cancers based on differences in the natural history of different cancers. We chose this milestone because it represents a highly pragmatic time point at which many survivors of cancer and their managing clinicians reexamine plans for cancer surveillance and general health maintenance. In support of the 5-year milestone, the longest running study of long-term survivors of cancer, to our knowledge, the Childhood Cancer Survivor Study, is limited entirely to patients who have survived 5 years from their cancer diagnosis.^49,50,51,52^ Last, although not the focus of this study, there can be a difference in risk profiles of patients before vs after 5 years from diagnosis. For example, we found that patients with ER-negative tumors were associated with higher cancer-specific mortality during the initial 5 years after diagnosis but with lower risk of cancer-specific mortality after 5 years, presumably because most ER-negative recurrences take place within 5 years.

### Limitations

There are several limitations to this study. First, validation of the models was performed through internal data validation only. Second, there were only a few clinical- and treatment-related factors in the SEER database. We included information about cancer stage and treatment at the time of diagnosis; however, we did not have information on the entire course of treatment, disease recurrence, or progression, which are crucial in estimating mortality for populations with cancer. Most important, data on patient comorbidities were not available, and therefore comorbidities could not be examined in our study. Variations in treatment and access to care can be significantly associated with cancer outcomes, particularly in racial and ethnic minority groups that often experience disparities in accessing quality health care services (eTable 2 in Supplement 1). Structural racism is likely associated with the disparities in treatment based on race and ethnicity, resulting in limited availability of specialized cancer treatments and support for these groups. However, this study could not assess treatment patterns or access to care due to data limitations.

## Conclusions

In obtaining cancer vs noncancer risk assessments, this cohort study stratified patients with cancer into 3 risk groups (low, intermediate, and high) of mortality based on cancer-specific prognostic factors that are associated with mortality. We found that the risk of cancer-specific vs noncancer-specific mortality varied substantially by cancer risk group, further informing the need for a personalized, risk-stratified approach to care that would eliminate unnecessary extended oncologic follow-up by optimizing the coordination between treating oncologists and PCPs. Future studies should include more follow-up information regarding treatment and disease recurrence.

## Notes

Supplement 1. **eTable 1.** Frequency Distribution of Noncancer Causes of Death in the Cancer Cohorts **eTable 2.** Crosstabulations of Race vs Income and Race vs Residence (Metropolitan) **eTable 3.** Estimates From LASSO for Breast Cancer Cohort, Prostate Cancer Cohort, and Colon Cancer Cohort **eFigure 1.** Survival Time Ratio of Noncancer Specific Mortality Among Patients With Breast, Prostate, Colon and Rectal Cancer **eFigure 2.** Cumulative Mortality for Patients With Breast, Prostate, Colon, and Rectal Cancer Who Were Categorized as Intermediate Risk
eMethods.

Supplement 2.
Data Sharing Statement

## References

[1] Mayer DK, Alfano CM. Personalized risk-stratified cancer follow-up care: its potential for healthier survivors, happier clinicians, and lower costs. J Natl Cancer Inst. 2019;111(5):442-448. doi:10.1093/jnci/djy232 30726949 PMC6804411

[2] Ye Y, Zheng Y, Miao Q, Ruan H, Zhang X. Causes of death among prostate cancer patients aged 40 years and older in the United States. Front Oncol. 2022;12:914875. doi:10.3389/fonc.2022.914875 35847902 PMC9286245

[3] Feng Y, Jin H, Guo K, Wasan HS, Ruan S, Chen C. Causes of death after colorectal cancer diagnosis: a population-based study. Front Oncol. 2021;11:647179. doi:10.3389/fonc.2021.647179 33859947 PMC8042257

[4] Abdel-Qadir H, Austin PC, Lee DS, . A population-based study of cardiovascular mortality following early-stage breast cancer. JAMA Cardiol. 2017;2(1):88-93. doi:10.1001/jamacardio.2016.3841 27732702

[5] Howlader N, Noone AM, Krapcho M, et al. SEER cancer statistics review, 1975-2012. National Cancer Institute. April 2015. Accessed February 1, 2023. https://seer.cancer.gov/csr/1975_2012/

[6] Alfano CM, Mayer DK, Bhatia S, . Implementing personalized pathways for cancer follow-up care in the United States: proceedings from an American Cancer Society–American Society of Clinical Oncology summit. CA Cancer J Clin. 2019;69(3):234-247. doi:10.3322/caac.21558 30849190 PMC7376887

[7] McCabe MS, Partridge AH, Grunfeld E, Hudson MM. Risk-based health care, the cancer survivor, the oncologist, and the primary care physician. Semin Oncol. 2013;40(6):804-812. doi:10.1053/j.seminoncol.2013.09.004 24331199 PMC4465133

[8] Oeffinger KC, McCabe MS. Models for delivering survivorship care. J Clin Oncol. 2006;24(32):5117-5124. doi:10.1200/JCO.2006.07.0474 17093273

[9] Hoon LS, Chi Sally CW, Hong-Gu H. Effect of psychosocial interventions on outcomes of patients with colorectal cancer: a review of the literature. Eur J Oncol Nurs. 2013;17(6):883-891. doi:10.1016/j.ejon.2013.05.001 23759360

[10] Roychoudhuri R, Evans H, Robinson D, Møller H. Radiation-induced malignancies following radiotherapy for breast cancer. Br J Cancer. 2004;91(5):868-872. doi:10.1038/sj.bjc.6602084 15292931 PMC2409877

[11] Forsythe LP, Parry C, Alfano CM, . Use of survivorship care plans in the United States: associations with survivorship care. J Natl Cancer Inst. 2013;105(20):1579-1587. doi:10.1093/jnci/djt258 24096621 PMC3797024

[12] Bluethmann SM, Mariotto AB, Rowland JH. Anticipating the “silver tsunami”: prevalence trajectories and comorbidity burden among older cancer survivors in the United States. Cancer Epidemiol Biomarkers Prev. 2016;25(7):1029-1036. doi:10.1158/1055-9965.EPI-16-0133 27371756 PMC4933329

[13] Leach CR, Weaver KE, Aziz NM, . The complex health profile of long-term cancer survivors: prevalence and predictors of comorbid conditions. J Cancer Surviv. 2015;9(2):239-251. doi:10.1007/s11764-014-0403-1 25319681

[14] Tremblay D, Touati N, Bilodeau K, Prady C, Usher S, Leblanc Y. Risk-stratified pathways for cancer survivorship care: insights from a deliberative multi-stakeholder consultation. Curr Oncol. 2021;28(5):3408-3419. doi:10.3390/curroncol28050295 34590587 PMC8482148

[15] Maher J, Petchey L, Greenfield D, Levitt G, Fraser M. Implementation of nationwide cancer survivorship plans: experience from the UK. J Cancer Policy. 2018;15(part B):76-81. doi:10.1016/j.jcpo.2018.01.002

[16] Chen Y, Zhang Y, Yang W, . Accuracy of a nomogram to predict the survival benefit of surgical axillary staging in T1 breast cancer patients. Medicine (Baltimore). 2018;97(26):e11273. doi:10.1097/MD.0000000000011273 29953003 PMC6039583

[17] Carroll R, Lawson AB, Jackson CL, Zhao S. Assessment of spatial variation in breast cancer–specific mortality using Louisiana SEER data. Soc Sci Med. 2017;193:1-7. doi:10.1016/j.socscimed.2017.09.045 28985516 PMC5659900

[18] Abdel-Rahman O. Assessment of the prognostic and discriminating value of the novel bioscore system for breast cancer; a SEER database analysis. Breast Cancer Res Treat. 2017;164(1):231-236. doi:10.1007/s10549-017-4244-2 28417332

[19] Yi M, Mittendorf EA, Cormier JN, . Novel staging system for predicting disease-specific survival in patients with breast cancer treated with surgery as the first intervention: time to modify the current American Joint Committee on Cancer staging system. J Clin Oncol. 2011;29(35):4654-4661. doi:10.1200/JCO.2011.38.3174 22084362 PMC3236648

[20] Chen HL, Zhou MQ, Tian W, Meng KX, He HF. Effect of age on breast cancer patient prognoses: a population-based study using the SEER 18 database. PLoS One. 2016;11(10):e0165409. doi:10.1371/journal.pone.0165409 27798652 PMC5087840

[21] Xu YB, Liu H, Cao QH, Ji JL, Dong RR, Xu D. Evaluating overall survival and competing risks of survival in patients with early-stage breast cancer using a comprehensive nomogram. Cancer Med. 2020;9(12):4095-4106. doi:10.1002/cam4.3030 32314546 PMC7300414

[22] Cai H, Zhang Y, Liu X, . Association of age and cause-special mortality in patients with stage I/ II colon cancer: a population-based competing risk analysis. PLoS One. 2020;15(10):e0240715. doi:10.1371/journal.pone.0240715 33064784 PMC7567365

[23] Rasul R, Golden A, Feuerstein MA. Prostate cancer risk group is associated with other-cause mortality in men with localized prostate cancer. Can Urol Assoc J. 2020;14(10):E507-E513. doi:10.5489/cuaj.6324 32432539 PMC7716825

[24] Daskivich TJ, Howard LE, Amling CL, . Competing risks of mortality among men with biochemical recurrence after radical prostatectomy. J Urol. 2020;204(3):511-517. doi:10.1097/JU.0000000000001036 32243242

[25] Hudson MM. A model for care across the cancer continuum. Cancer. 2005;104(11)(suppl):2638-2642. doi:10.1002/cncr.21250 16258932

[26] Institute of Medicine and National Research Council. From Cancer Patient to Cancer Survivor: Lost in Transition. The National Academies Press; 2006:534.

[27] National Cancer Institute; Surveillance, Epidemiology, and End Results Program. Overview of the SEER program. Accessed February 1, 2023. https://seer.cancer.gov/about/overview.html

[28] National Cancer Institute; Surveillance, Epidemiology, and End Results Program. SEER cause of death recode 1969+ (04/16/2102). 2015. Accessed February 1, 2023. https://seer.cancer.gov/codrecode/1969_d04162012/index.html

[29] Rowland JH, Gallicchio L, Mollica M, Saiontz N, Falisi AL, Tesauro G. Survivorship science at the NIH: lessons learned from grants funded in fiscal year 2016. J Natl Cancer Inst. 2019;111(2):109-117. doi:10.1093/jnci/djy208 30657942 PMC6657281

[30] Alam TF, Rahman MS, Bari W. On estimation for accelerated failure time models with small or rare event survival data. BMC Med Res Methodol. 2022;22(1):169. doi:10.1186/s12874-022-01638-1 35689190 PMC9188212

[31] Cleves M, Gould W, Gould WW, Gutierrez R, Marchenko Y. An Introduction to Survival Analysis Using Stata. Stata Press; 2008.

[32] Montaseri M, Charati JY, Espahbodi F. Application of parametric models to a survival analysis of hemodialysis patients. Nephrourol Mon. 2016;8(6):e28738. doi:10.5812/numonthly.28738 27896235 PMC5120235

[33] Johansson ALV, Trewin CB, Hjerkind KV, Ellingjord-Dale M, Johannesen TB, Ursin G. Breast cancer–specific survival by clinical subtype after 7 years follow-up of young and elderly women in a nationwide cohort. Int J Cancer. 2019;144(6):1251-1261. doi:10.1002/ijc.31950 30367449

[34] Nelson DR, Brown J, Morikawa A, Method M. Breast cancer–specific mortality in early breast cancer as defined by high-risk clinical and pathologic characteristics. PLoS One. 2022;17(2):e0264637. doi:10.1371/journal.pone.0264637 35213669 PMC8880870

[35] Shih HJ, Fang SC, An L, Shao YJ. Early-onset prostate cancer is associated with increased risks of disease progression and cancer-specific mortality. Prostate. 2021;81(2):118-126. doi:10.1002/pros.24087 33152137

[36] Gnerlich JL, Deshpande AD, Jeffe DB, Sweet A, White N, Margenthaler JA. Elevated breast cancer mortality in women younger than age 40 years compared with older women is attributed to poorer survival in early-stage disease. J Am Coll Surg. 2009;208(3):341-347. doi:10.1016/j.jamcollsurg.2008.12.001 19317994 PMC3262236

[37] Han JG, Jiang YD, Zhang CH, . Clinicopathologic characteristics and prognosis of young patients with breast cancer. Breast. 2011;20(4):370-372. doi:10.1016/j.breast.2011.02.011 21392995

[38] Burdett N, Vincent AD, O’Callaghan M, Kichenadasse G. Competing risks in older patients with cancer: a systematic review of geriatric oncology trials. J Natl Cancer Inst. 2018;110(8):825-830. doi:10.1093/jnci/djy111 30011032

[39] Hanrahan EO, Gonzalez-Angulo AM, Giordano SH, . Overall survival and cause-specific mortality of patients with stage T1a,bN0M0 breast carcinoma. J Clin Oncol. 2007;25(31):4952-4960. doi:10.1200/JCO.2006.08.0499 17971593

[40] Grann V, Troxel AB, Zojwalla N, Hershman D, Glied SA, Jacobson JS. Regional and racial disparities in breast cancer–specific mortality. Soc Sci Med. 2006;62(2):337-347. doi:10.1016/j.socscimed.2005.06.038 16051406

[41] Nasrazadani A, Marti JLG, Kip KE, . Breast cancer mortality as a function of age. Aging (Albany NY). 2022;14(3):1186-1199. doi:10.18632/aging.203881 35134749 PMC8876898

[42] Hendrick RE, Monticciolo DL, Biggs KW, Malak SF. Age distributions of breast cancer diagnosis and mortality by race and ethnicity in US women. Cancer. 2021;127(23):4384-4392. doi:10.1002/cncr.33846 34427920

[43] Kim HJ, Kim S, Freedman RA, Partridge AH. The impact of young age at diagnosis (age <40 years) on prognosis varies by breast cancer subtype: a U.S. SEER database analysis. Breast. 2022;61:77-83. doi:10.1016/j.breast.2021.12.006 34923225 PMC8693310

[44] Tao L, Schwab RB, San Miguel Y, . Breast cancer mortality in older and younger patients in California. Cancer Epidemiol Biomarkers Prev. 2019;28(2):303-310. doi:10.1158/1055-9965.EPI-18-0353 30333222 PMC6363871

[45] San Miguel Y, Gomez SL, Murphy JD, . Age-related differences in breast cancer mortality according to race/ethnicity, insurance, and socioeconomic status. BMC Cancer. 2020;20(1):228. doi:10.1186/s12885-020-6696-8 32178638 PMC7076958

[46] van de Water W, Markopoulos C, van de Velde CJ, . Association between age at diagnosis and disease-specific mortality among postmenopausal women with hormone receptor–positive breast cancer. JAMA. 2012;307(6):590-597. doi:10.1001/jama.2012.84 22318280

[47] Siegelmann-Danieli N, Khandelwal V, Wood GC, . Breast cancer in elderly women: outcome as affected by age, tumor features, comorbidities, and treatment approach. Clin Breast Cancer. 2006;7(1):59-66. doi:10.3816/CBC.2006.n.014 16764745

[48] Smith T, Stein KD, Mehta CC, . The rationale, design, and implementation of the American Cancer Society’s studies of cancer survivors. Cancer. 2007;109(1):1-12. doi:10.1002/cncr.22387 17146781

[49] Frobisher C, Glaser A, Levitt GA, . Risk stratification of childhood cancer survivors necessary for evidence-based clinical long-term follow-up. Br J Cancer. 2017;117(11):1723-1731. doi:10.1038/bjc.2017.347 29065109 PMC5729444

[50] Henderson TO, Liu Q, Turcotte LM, . Association of changes in cancer therapy over 3 decades with risk of subsequent breast cancer among female childhood cancer survivors: a report from the Childhood Cancer Survivor Study (CCSS). JAMA Oncol. 2022;8(12):1765-1774. doi:10.1001/jamaoncol.2022.4649 36227603 PMC9562103

[51] Suh E, Stratton KL, Leisenring WM, . Late mortality and chronic health conditions in long-term survivors of early-adolescent and young adult cancers: a retrospective cohort analysis from the Childhood Cancer Survivor Study. Lancet Oncol. 2020;21(3):421-435. doi:10.1016/S1470-2045(19)30800-9 32066543 PMC7392388

[52] Green DM, Nolan VG, Goodman PJ, . The cyclophosphamide equivalent dose as an approach for quantifying alkylating agent exposure: a report from the Childhood Cancer Survivor Study. Pediatr Blood Cancer. 2014;61(1):53-67. doi:10.1002/pbc.24679 23940101 PMC3933293

