# Supplementary material for: Relative Burden of Cancer and Noncancer Mortality Among Long-Term Survivors of Breast, Prostate, and Colorectal Cancer in the US
Source: JAMA Netw Open. 2023 Jul 12;6(7):e2323115. doi: 10.1001/jamanetworkopen.2023.23115 (PMC10339147; doi:10.1001/jamanetworkopen.2023.23115)
Supplement: Supplement 1. — eTable 1. Frequency Distribution of Noncancer Causes of Death in the Cancer Cohorts eTable 2. Crosstabulations of Race vs Income and Race vs Residence (Metropolitan) eTable 3. Estimates From LASSO for Breast Cancer Cohort, Prostate Cancer Cohort, and Colon Cancer Cohort eFigure 1. Survival Time Ratio of Noncancer Specific Mortality Among Patients With Breast, Prostate, Colon and Rectal Cancer eFigure 2. Cumulative Mortality for Patients With Breast, Prostate, Colon, and Rectal Cancer Who Were Categorized as Intermediate Risk eMethods. [file jamanetwopen-e2323115-s001.pdf]

# Supplementary Online Content

KC M, Fan J, Hyslop T, et al. Relative burden of cancer and noncancer mortality among long-term survivors of breast, prostate, and colorectal cancer in the US. *JAMA Netw Open.* 2023;6(7):e2323115. doi:10.1001/jamanetworkopen.2023.23115

**eTable 1.** Frequency Distribution of Noncancer Causes of Death in the Cancer Cohorts

**eTable 2.** Crosstabulations of Race vs Income and Race vs Residence (Metropolitan)

**eTable 3.** Estimates From LASSO for Breast Cancer Cohort, Prostate Cancer Cohort**,** and Colon Cancer Cohort

**eFigure 1.** Survival Time Ratio of Noncancer Specific Mortality Among Patients With Breast, Prostate, Colon and Rectal Cancer

**eFigure 2.** Cumulative Mortality for Patients With Breast, Prostate, Colon, and Rectal Cancer Who Were Categorized as Intermediate Risk

**eMethods.**

This supplementary material has been provided by the authors to give readers additional information about their work.

© 2023 KC M et al. *JAMA Network Open.*

**eTable 1.** Frequency Distribution of Noncancer Causes of Death in the Cancer Cohorts

| SN | Non-cancer specific causes of death | N | % |
|---|---|---|---|
| | **Breast Cancer Cohort** | | |
| **SN** | **Non-cancer specific causes of death** | **N** | **%** |
| 1 | Diseases of Heart | 9210 | 24.02 |
| 2 | Other Cause of Death | 7073 | 18.44 |
| 3 | Alzheimers (ICD-9 and 10 only) | 2727 | 7.11 |
| 4 | Cerebrovascular Diseases | 2522 | 6.58 |
| 5 | Chronic Obstructive Pulmonary Disease and Allied Cond | 2474 | 6.45 |
| 6 | Lung and Bronchus | 1894 | 4.94 |
| 7 | Accidents and Adverse Effects | 1025 | 2.67 |
| 8 | Diabetes Mellitus | 978 | 2.55 |
| 9 | Pneumonia and Influenza | 976 | 2.55 |
| 10 | Pancreas | 738 | 1.92 |
| | **Prostate Cancer Cohort** | | |
| **SN** | **Non-cancer specific causes of death** | **N** | **%** |
| 1 | Diseases of Heart | 1758 | 24.49 |
| 2 | Other Cause of Death | 1156 | 16.1 |
| 3 | Lung and Bronchus | 455 | 6.34 |
| 4 | Chronic Obstructive Pulmonary Disease and Allied Cond | 441 | 6.14 |
| 5 | Cerebrovascular Diseases | 343 | 4.78 |
| 6 | Alzheimers (ICD-9 and 10 only) | 249 | 3.47 |
| 7 | Accidents and Adverse Effects | 243 | 3.38 |
| 8 | Diabetes Mellitus | 210 | 2.93 |
| 9 | Pancreas | 195 | 2.72 |
| 10 | Nephritis, Nephrotic Syndrome and Nephrosis | 144 | 2.01 |
| | **Colon Cancer Cohort** | | |
| **SN** | **Non-cancer specific causes of death** | **N** | **%** |
| 1 | Diseases of Heart | 6690 | 28.96 |
| 2 | Other Cause of Death | 3751 | 16.24 |
| 3 | Alzheimers (ICD-9 and 10 only) | 1561 | 6.76 |
| 4 | Cerebrovascular Diseases | 1460 | 6.32 |
| 5 | Chronic Obstructive Pulmonary Disease and Allied Cond | 1406 | 6.09 |
| 6 | Lung and Bronchus | 956 | 4.14 |
| 7 | Diabetes Mellitus | 835 | 3.61 |
| 8 | Pneumonia and Influenza | 666 | 2.88 |
| 9 | Accidents and Adverse Effects | 596 | 2.58 |
| 10 | Nephritis, Nephrotic Syndrome and Nephrosis | 576 | 2.49 |
| | **Rectal Cancer Cohort** | | |
| **SN** | **Non-cancer specific causes of death** | **N** | **%** |
| 1 | Diseases of Heart | 1710 | 27.51 |
| 2 | Other Cause of Death | 979 | 15.75 |
| 3 | Chronic Obstructive Pulmonary Disease and Allied Cond | 391 | 6.29 |

© 2023 KC M et al. *JAMA Network Open.*

| | | | |
|---|---|---|---|
| 4 | Cerebrovascular Diseases | 351 | 5.65 |
| 5 | Lung and Bronchus | 348 | 5.6 |
| 6 | Alzheimers (ICD-9 and 10 only) | 296 | 4.76 |
| 7 | Diabetes Mellitus | 219 | 3.52 |
| 8 | Accidents and Adverse Effects | 169 | 2.72 |
| 9 | Pneumonia and Influenza | 154 | 2.48 |
| 10 | Nephritis, Nephrotic Syndrome and Nephrosis | 150 | 2.41 |

© 2023 KC M et al. *JAMA Network Open.*

**eTable 2.** Crosstabulations of Race vs Income and Race vs Residence (Metropolitan)

| Race/ethnicity | Income | | | | |
|---|---|---|---|---|---|
| | **Overall** | **< $40,000** | **$40,000 - $54,999** | **$55,000 - $69,999** | **$70,000+** |
| **NH- white** | 446058 (71.06) | 16555 (3.71) | 83625 (18.75) | 163063 (36.56) | 182815 (40.98) |
| **NH-Black** | 64117 (10.21) | 5006(7.81) | 16376 (25.54) | 26102 (40.71) | 16633 (25.94) |
| **NH-Asian** | 50719 (8.08) | 88 (0.17) | 1472 (2.90) | 17862 (35.22) | 31297 (61.71) |
| **Hispanic** | 64200 (10.23) | 630 (0.98) | 8840 (13.77) | 33173 (51.67) | 21557 (33.58) |
| **NH-American Indian** | 2608 (0.42) | 214 (8.21) | 750 (28.76) | 901 (34.55) | 743 (28.49) |

Note: There was an association between race/ethnicity and income (p-value<0.01).

| Race/ethnicity | Residence | | |
|---|---|---|---|
| | **Overall** | **Non-metropolitan** | **Metropolitan** |
| **NH-White** | 446058 (71.06) | 61383 (13.76) | 384675 (86.24) |
| **NH-Black** | 64117 (10.21) | 5779 (9.01) | 58338 (90.99) |
| **NH-Asian** | 50719 (8.08) | 1700 (3.35) | 49019 (96.65) |
| **Hispanic** | 64200 (10.23) | 2349 (3.66) | 61851 (96.34) |
| **NH-American Indian** | 2608 (0.42) | 584 (22.39) | 2024 (77.61) |

Note: There was an association between race/ethnicity and residence (p-value<0.01).

© 2023 KC M et al. *JAMA Network Open.*

**eTable 3a.** Estimates From LASSO for Breast Cancer Cohort

| Breast specific mortality | | Non-breast specific mortality | |
|---|---|---|---|
| **Predictors** | **Coefficients** | **Predictors** | **Coefficients** |
| Stage III | 1.537251072 | Age: 65+ | 2.699201705 |
| Stage II | 0.720426478 | Age: 60-64 | 1.439952426 |
| Grade III | 0.684887731 | Age: 55-59 | 0.988162494 |
| Grade II | 0.534389507 | Stage III | 0.636884153 |
| Age: 65+ | 0.465242924 | Age: 50 to 54 | 0.567543968 |
| ER Positive | 0.460846144 | Stage II | 0.275906812 |
| Node positive | 0.32159283 | Income: <$40,000 | 0.268716028 |
| Race: NH Black | 0.272865599 | Race: NH American Indian | 0.231050449 |
| Income: <$40,000 | 0.210125129 | Income: $40,000-$54,999 | 0.169862759 |
| Income: $40,000-$54,999 | 0.151766213 | Race: NH Black | 0.162013621 |
| Race: NH American Indian | 0.109816262 | Grade III | 0.063483896 |
| Income: $55,000-$69,999 | 0.106816932 | Income: $55,000-$69,999 | 0.029468223 |
| Age: 60-64 | 0.090598634 | Grade II | 0.021965937 |
| Race: Hispanic | 0.081964288 | Laterality: left | -0.003487806 |
| Age: 55-59 | 0.04018637 | PR Positive | -0.016875338 |
| Laterality: left | 0.029488998 | Diagnosis year2005 | -0.022470294 |
| Received chemotherapy | 0 | Metropolitan | -0.022943673 |
| Metropolitan | 0 | ER Positive | -0.02684002 |
| Age: 50 to 54 | -0.047973637 | Diagnosis year2004 | -0.027342957 |
| Received radiation | -0.113938681 | Node positive | -0.032457425 |
| PR Positive | -0.12279612 | Diagnosis year2006 | -0.05338106 |
| Diagnosis year2004 | -0.132400159 | Diagnosis year2007 | -0.053936603 |
| Race: NH Asian | -0.13352793 | Diagnosis year2009 | -0.098477185 |
| Diagnosis year2006 | -0.165020348 | Diagnosis year2008 | -0.102628064 |
| Diagnosis year2005 | -0.182476773 | Diagnosis year2010 | -0.1055015 |
| Diagnosis year2011 | -0.216875428 | Race: Hispanic | -0.128755726 |
| Diagnosis year2008 | -0.220891581 | Diagnosis year2011 | -0.147772815 |
| Diagnosis year2007 | -0.230069019 | Diagnosis year2012 | -0.189877755 |
| Diagnosis year2010 | -0.260495116 | Diagnosis year2013 | -0.195374001 |
| Diagnosis year2009 | -0.288913878 | Diagnosis year2014 | -0.229486158 |
| Diagnosis year2012 | -0.314935441 | Received radiation | -0.288273589 |
| Diagnosis year2014 | -0.336115864 | Race: NH Asian | -0.308808776 |
| Diagnosis year2013 | -0.354917735 | Received chemotherapy | -0.522345065 |

© 2023 KC M et al. *JAMA Network Open.*

**eTable 3b.** Estimates From LASSO for Prostate Cancer Cohort

| Prostate specific mortality | | Non-prostate specific mortality | |
|---|---|---|---|
| **Predictors** | **Coefficients** | **Predictors** | **Coefficients** |
| Gleason score >=8 | 2.034374852 | Age: 65+ | 1.974076105 |
| PSA >20 ng/ml | 0.992756164 | Age: 60-64 | 1.085337557 |
| Gleason score 7 | 0.959798764 | Age: 55-59 | 0.785334807 |
| Received chemotherapy | 0.842982458 | Gleason score >=8 | 0.593229466 |
| Age: 65+ | 0.786030325 | PSA >20 ng/ml | 0.48315396 |
| Diagnosis year2005 | 0.576025911 | Income: <$40,000 | 0.472943656 |
| PSA 10-20 ng/ml | 0.555856856 | Age: 50 to 54 | 0.40508499 |
| Diagnosis year2007 | 0.512294762 | PSA 10-20 ng/ml | 0.37328792 |
| Diagnosis year2006 | 0.46865235 | Income: $40,000-$54,999 | 0.308611712 |
| Race: NH American Indian | 0.301474953 | Gleason score 7 | 0.307532718 |
| Age: 60-64 | 0.299958217 | Diagnosis year2008 | 0.160165023 |
| Age: 55-59 | 0.229695509 | Race: NH Black | 0.158281607 |
| Stage III | 0.194229898 | Diagnosis year2009 | 0.139001752 |
| Income: <$40,000 | 0.178404795 | Income: $55,000-$69,999 | 0.085547563 |
| Income: $55,000-$69,999 | 0.109471677 | Diagnosis year2006 | 0.056766055 |
| Diagnosis year2008 | 0.09756749 | Diagnosis year2007 | 0.023847414 |
| Race: NH Black | 0.078537701 | Received chemotherapy | 0 |
| Age: 50 to 54 | 0.017222436 | Metropolitan | 0 |
| Income: $40,000-$54,999 | 0.000509206 | Diagnosis year2010 | -0.067855622 |
| Metropolitan | 0 | Diagnosis year2011 | -0.083717596 |
| Race: Hispanic | -0.017384104 | Diagnosis year2012 | -0.096460298 |
| Stage II | -0.22066363 | Race: Hispanic | -0.134747387 |
| Diagnosis year2009 | -0.284852766 | Diagnosis year2013 | -0.203359358 |
| Race: NH Asian | -0.45112247 | Diagnosis year2005 | -0.234171545 |
| Diagnosis year2012 | -0.675802517 | Stage II | -0.241639917 |
| Diagnosis year2010 | -0.696123364 | Race: NH Asian | -0.245438383 |
| Diagnosis year2014 | -0.715420816 | Diagnosis year2014 | -0.271951938 |
| Diagnosis year2011 | -0.723133848 | Race: NH American Indian | -0.448841961 |
| Diagnosis year2013 | -0.794248077 | Stage III | -0.58350858 |

© 2023 KC M et al. *JAMA Network Open.*

**eTable 3c.** Estimates From LASSO for Colon Cancer Cohort

| **Colon specific mortality** | | **Non-colon specific mortality** | |
|---|---|---|---|
| Predictors | Coefficients | Predictors | Coefficients |
| Stage III | 1.099262148 | Age: 65+ | 2.475938507 |
| Received radiation | 0.678781844 | Age: 60-64 | 1.286602288 |
| Stage II | 0.655246821 | Age: 55-59 | 0.921193796 |
| Age: 65+ | 0.50581014 | Age: 50 to 54 | 0.44765914 |
| Sex: Male | 0.237220064 | Stage III | 0.230463958 |
| Race: NH Black | 0.230035243 | Received radiation | 0.220824173 |
| Age: 60-64 | 0.162084578 | Stage II | 0.176699139 |
| Race: NH American Indian | 0.138694827 | Grade III | 0.158286473 |
| Received chemotherapy | 0.112573668 | Income: $40,000-$54,999 | 0.104610755 |
| Grade II | 0.102515375 | Sex: Male | 0.103691861 |
| Age: 55-59 | 0.086135265 | Grade II | 0.049579938 |
| Race: Hispanic | 0.07740056 | Income: <$40,000 | 0.040602506 |
| Age: 50 to 54 | 0.001206978 | Income: $55,000-$69,999 | 0.015514833 |
| Income: <$40,000 | 0 | Metropolitan | 0 |
| Income: $40,000-$54,999 | 0 | Diagnosis year2004 | 0 |
| Income: $55,000-$69,999 | 0 | Diagnosis year2005 | 0 |
| Diagnosis year2004 | 0 | Diagnosis year2006 | 0 |
| Diagnosis year2005 | 0 | Diagnosis year2007 | 0 |
| Diagnosis year2006 | 0 | Diagnosis year2008 | 0 |
| Diagnosis year2007 | 0 | Diagnosis year2009 | 0 |
| Diagnosis year2008 | 0 | Diagnosis year2010 | 0 |
| Diagnosis year2009 | 0 | Diagnosis year2011 | 0 |
| Diagnosis year2010 | 0 | Diagnosis year2012 | 0 |
| Diagnosis year2011 | 0 | Diagnosis year2013 | 0 |
| Diagnosis year2012 | 0 | Diagnosis year2014 | 0 |
| Diagnosis year2013 | 0 | Node positive | 0 |
| Diagnosis year2014 | 0 | Race: NH Black | -0.077714536 |
| Node positive | 0 | Race: NH American Indian | -0.201364532 |
| Grade III | -0.002445428 | Race: Hispanic | -0.220277134 |
| Race: NH Asian | -0.067874607 | Race: NH Asian | -0.379184944 |
| Metropolitan | -0.095197818 | Received chemotherapy | -0.607521451 |

© 2023 KC M et al. *JAMA Network Open.*

**eTable 3d.** Estimates From LASSO for Colon Cancer Cohort

| Rectal Specific Mortality | | Non-rectal specific mortality | |
|---|---|---|---|
| **Predictors** | **Coefficients** | **Predictors** | **Coefficients** |
| Stage III | 0.767787428 | Age: 65+ | 2.489941 |
| Age: 65+ | 0.489828926 | Age: 60-64 | 1.440028 |
| Race: NH American Indian | 0.440062967 | Age: 55-59 | 1.027247 |
| Stage II | 0.405510409 | Age: 50 to 54 | 0.58245 |
| Race: NH Black | 0.276920301 | Stage II | 0.22218 |
| Received radiation | 0.248965583 | Income: $40,000-$54,999 | 0.185543 |
| Income: <$40,000 | 0.233499737 | Stage III | 0.173978 |
| Age: 60-64 | 0.201605727 | Race: NH American Indian | 0.159218 |
| Sex: Male | 0.157909355 | Sex: Male | 0.153263 |
| Income: $55,000-$69,999 | 0.145678625 | Income: <$40,000 | 0.145227 |
| Income: $40,000-$54,999 | 0.145542349 | Income: $55,000-$69,999 | 0.067393 |
| Grade III | 0.136721223 | Received radiation | 0 |
| Race: Hispanic | 0.132288965 | Metropolitan | 0 |
| Grade II | 0.113521841 | Diagnosis year2004 | 0 |
| Received chemotherapy | 0.08699774 | Diagnosis year2005 | 0 |
| Age: 55-59 | 0.050525555 | Diagnosis year2006 | 0 |
| Age: 50 to 54 | 0.026208036 | Diagnosis year2007 | 0 |
| Diagnosis year2010 | 0.024210606 | Diagnosis year2008 | 0 |
| Node positive | 0 | Diagnosis year2009 | 0 |
| Diagnosis year2004 | -0.003333617 | Diagnosis year2010 | 0 |
| Race: NH Asian | -0.017040191 | Diagnosis year2011 | 0 |
| Metropolitan | -0.018932207 | Diagnosis year2012 | 0 |
| Diagnosis year2006 | -0.053334985 | Diagnosis year2013 | 0 |
| Diagnosis year2009 | -0.061803173 | Diagnosis year2014 | 0 |
| Diagnosis year2007 | -0.080438055 | Node positive | 0 |
| Diagnosis year2005 | -0.089145029 | Grade II | 0 |
| Diagnosis year2013 | -0.098261766 | Grade III | 0 |
| Diagnosis year2011 | -0.10421356 | Race: NH Black | -0.03386 |
| Diagnosis year2014 | -0.131655872 | Race: Hispanic | -0.18415 |
| Diagnosis year2012 | -0.1339987 | Received chemotherapy | -0.30955 |
| Diagnosis year2008 | -0.18215978 | Race: NH Asian | -0.36205 |

© 2023 KC M et al. *JAMA Network Open.*

**eFigure 1.** Survival Time Ratio of Noncancer Specific Mortality Among Patients With Breast, Prostate, Colon and Rectal Cancer

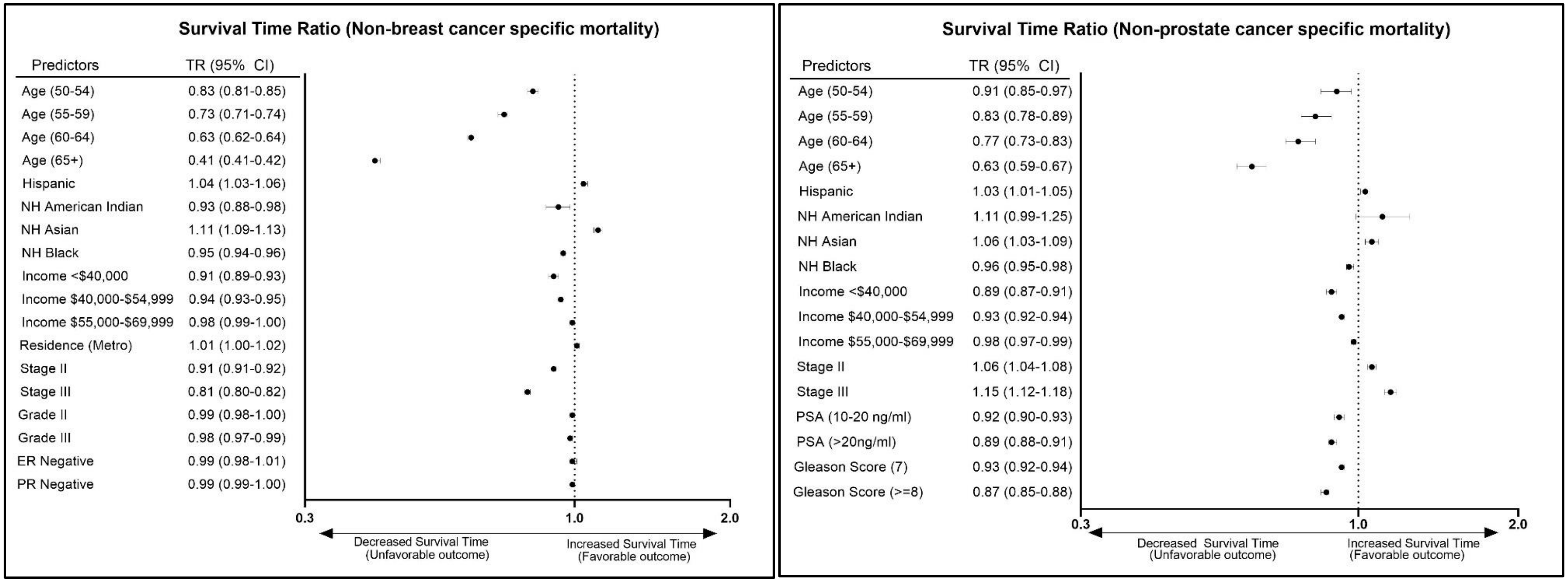


© 2023 KC M et al. *JAMA Network Open.*

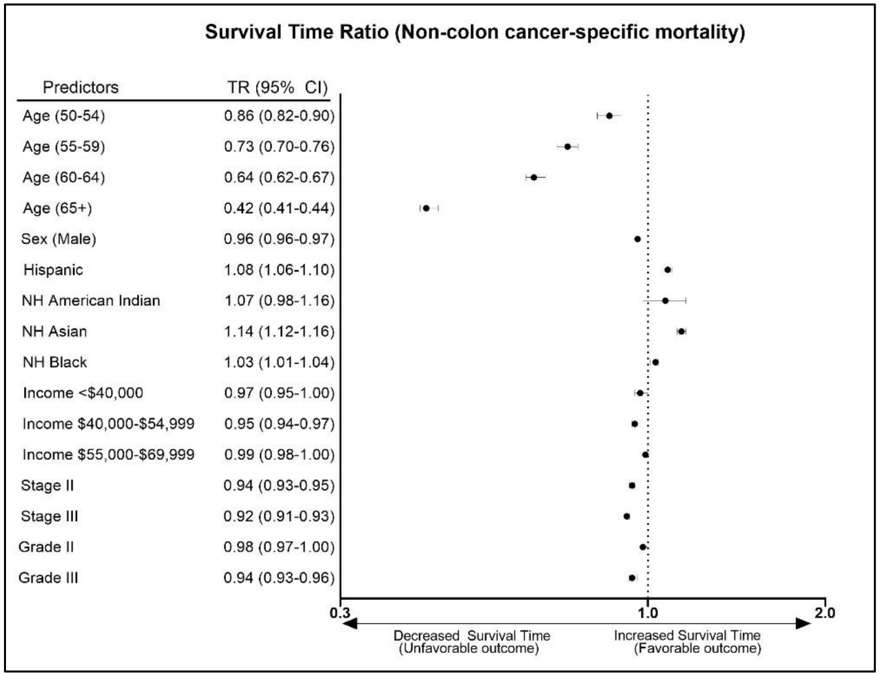


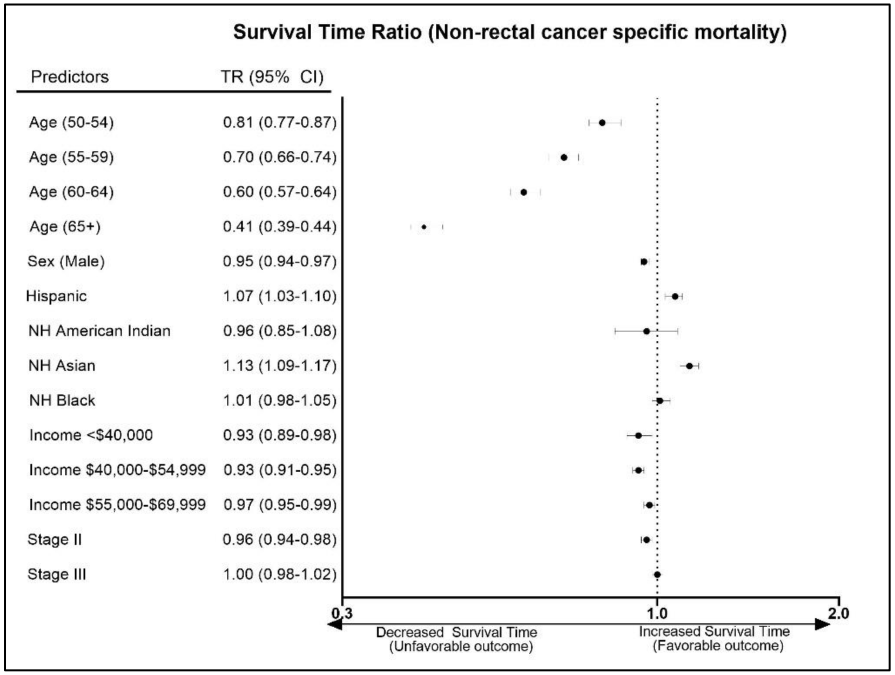


© 2023 KC M et al. *JAMA Network Open.*

**eFigure 2.** Cumulative Mortality for Patients With Breast, Prostate, Colon, and Rectal Cancer Who Were Categorized as Intermediate Risk

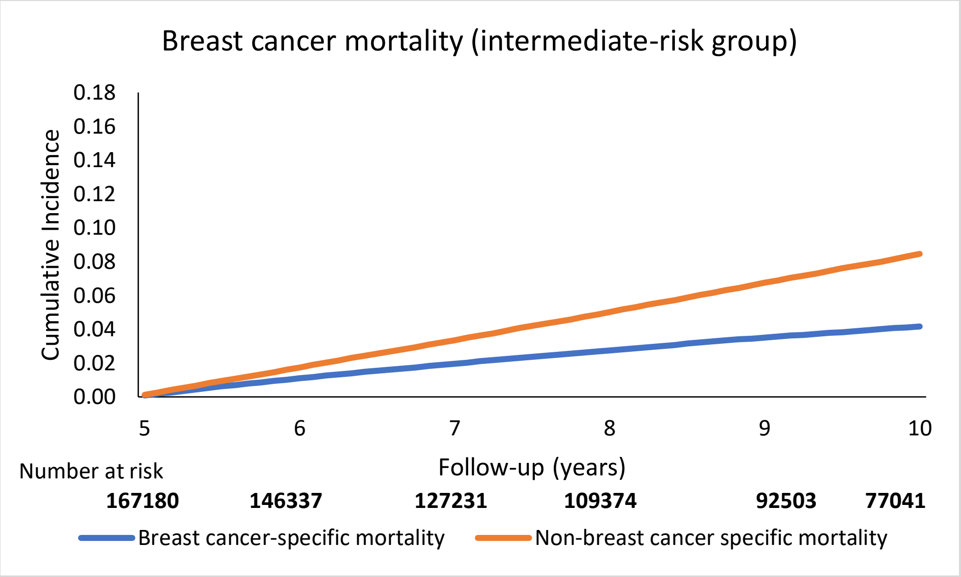


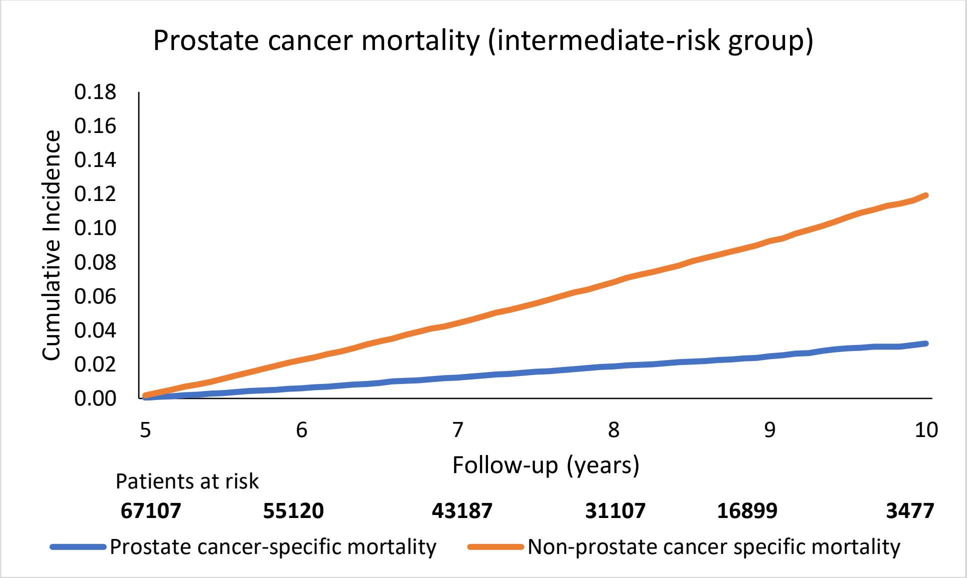


© 2023 KC M et al. *JAMA Network Open.*

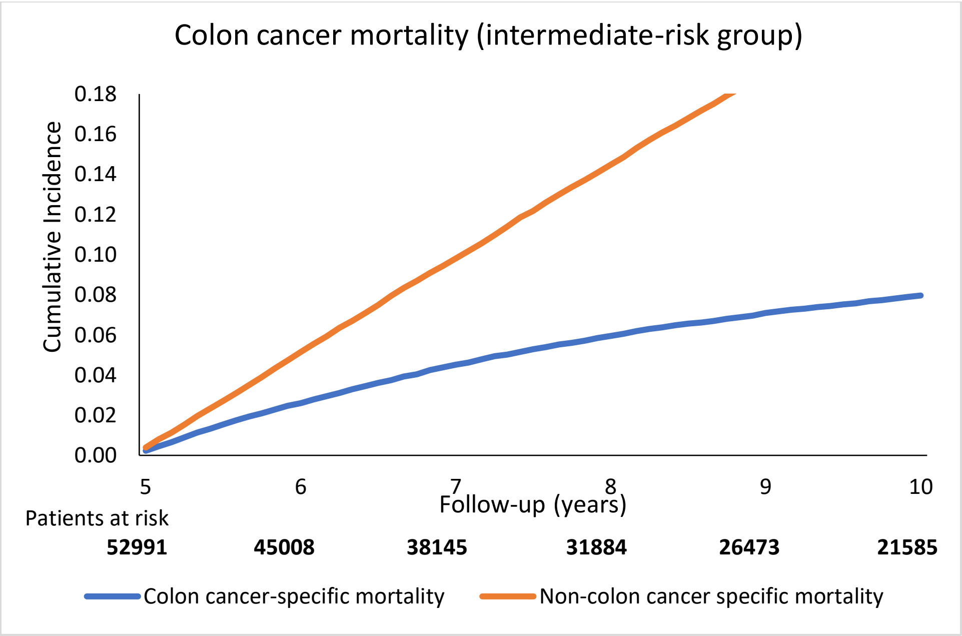


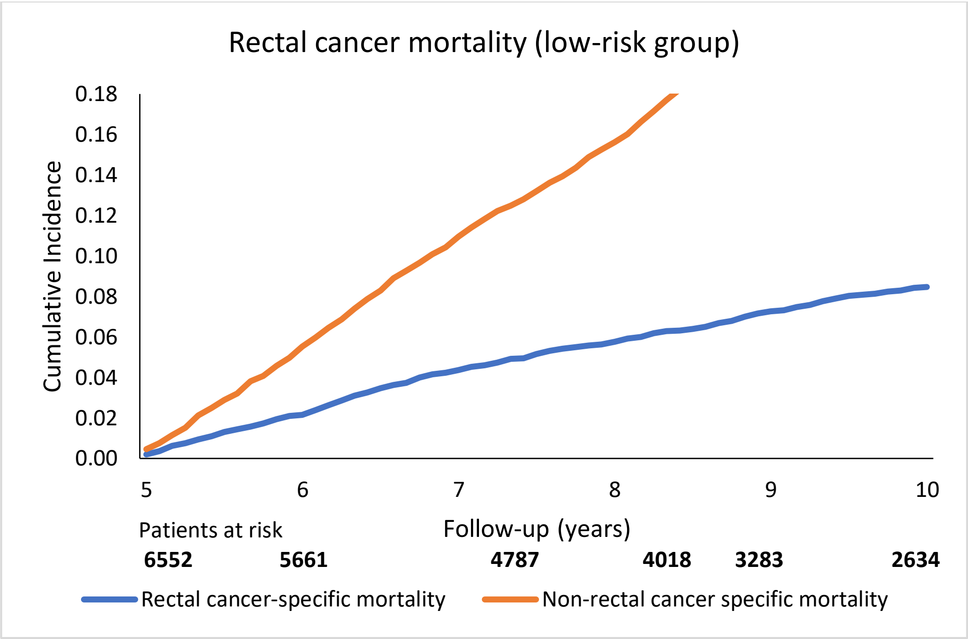


© 2023 KC M et al. *JAMA Network Open*.

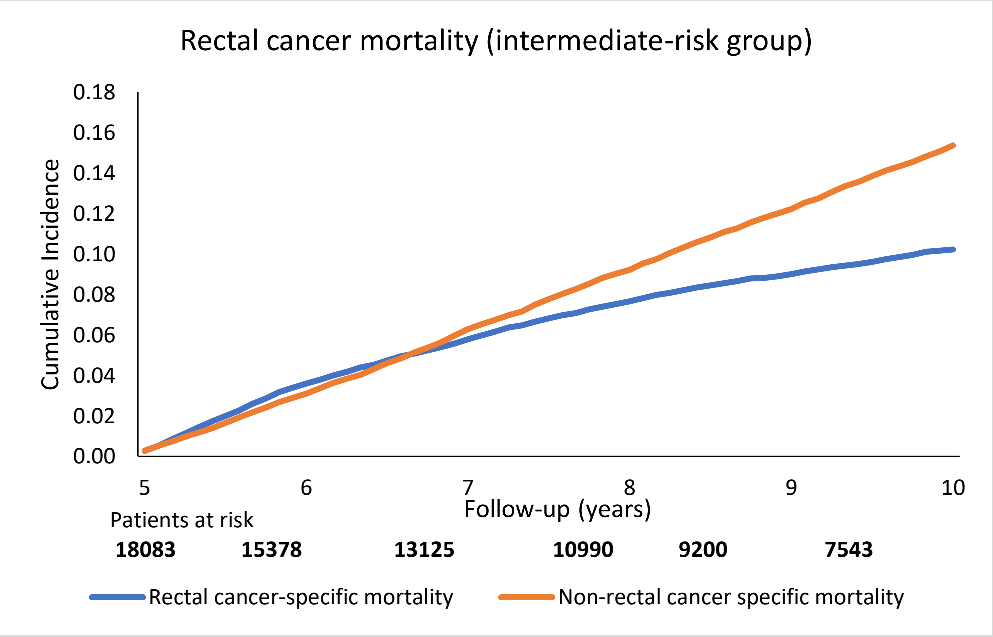


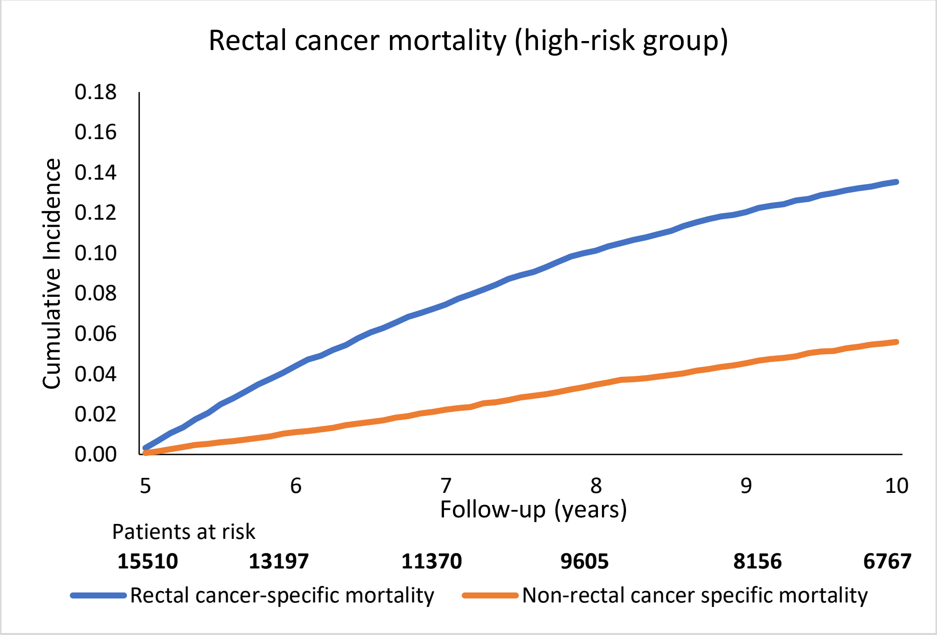


© 2023 KC M et al. *JAMA Network Open.*

**eMethods.**

**Exclusion of patients with missing information on demographic or clinical factors**

In our study, we excluded patients with missing information on demographic or clinical factors as well as those with missing follow-up time. This exclusion was necessary due to the nature of our study's goal, which is to accurately stratify patients into three risk categories: low, intermediate, and high-risk. To achieve this, it was crucial to have complete information on all variables included in the model.

Additionally, we employed LASSO regularization to select important variables and mitigate the risk of model overfitting. This regularization techniques requires non-missing values to perform the selection process effectively.

About 32% of patients were excluded due to missing demographic or clinical information or missing follow-up time. Notably, a significant portion of the excluded patients (77%) were prostate cancer patients. This high proportion is attributed to the limited availability of Gleason scores in the SEER database.

© 2023 KC M et al. *JAMA Network Open*.
