# Supplementary material for: Relative Burden of Cancer and Noncancer Mortality Among Long-Term Survivors of Breast, Prostate, and Colorectal Cancer in the US
Source: JAMA Netw Open. 2023 Jul 12;6(7):e2323115. doi: 10.1001/jamanetworkopen.2023.23115 (PMC10339147; doi:10.1001/jamanetworkopen.2023.23115)
Supplement: Supplement 2. — Data Sharing Statement [file jamanetwopen-e2323115-s002.pdf]

Data Sharing Statement

KC. Relative Burden of Cancer and Noncancer Mortality Among Long-Term Survivors of Breast, Prostate, and Colorectal Cancer in the US. *JAMA Netw Open*. Published July 12, 2023. doi:10.1001/jamanetworkopen.2023.23115

Data
Data available: No
Explanation for why data not available: No original data were generated or collected as part of this study. The SEER data used in this study are available from the National Cancer Institute.
